# Oral Passive Immunization With Plasma-Derived Polyreactive Secretory-Like IgA/M Partially Protects Mice Against Experimental Salmonellosis

**DOI:** 10.3389/fimmu.2018.02970

**Published:** 2018-12-18

**Authors:** Blaise Corthésy, Justine Monnerat, Marius Lötscher, Cédric Vonarburg, Alexander Schaub, Gilles Bioley

**Affiliations:** ^1^R&D Laboratory, Division of Immunology and Allergy, Lausanne University Hospital (CHUV), Épalinges, Switzerland; ^2^Research Department, CSL Behring AG, Bern, Switzerland

**Keywords:** passive immunization, secretory IgA, secretory IgM, *Salmonella*, protection

## Abstract

Secretory immunoglobulins have a critical role in defense of the gastrointestinal tract and are known to act by preventing bacterial acquisition. A stringent murine model of bacterial infection with *Salmonella enterica* Typhimurium was used to examine protection mediated by oral passive immunization with human plasma-derived polyreactive IgA and IgM antibodies (Abs) reconstituted as secretory-like immunoglobulins (SCIgA/M). This reagent has been shown to trigger *Salmonella* agglutination and to limit the entry of bacterium into intestinal Peyer's patches via immune exclusion. We now demonstrate that upon administration into ligated intestinal loops, SCIgA/M properly anchors in the mucus and is protected from degradation to a better extent that IgA/M or IgG. Moreover, prophylactic oral administration of SCIgA/M before intragastric infection of mice with a virulent strain of *S. enterica* Typhimurium allows to protect infected animals, as reflected by reduced colonization of both mucosal and systemic compartments, and conserved integrity of intestinal tissues. In comparison with IgA/M or IgG administration, SCIgA/M provided the highest degree of protection. Moreover, such protective efficacy is also observed after therapeutic oral delivery of SCIgA/M. Either prophylactic or therapeutic treatment with passively delivered SCIgA/M ensured survival of up to 50% of infected mice, while untreated animals all died. Our findings unravel the potential of oral passive immunization with plasma-derived polyreactive SCIgA/M Abs to fight gastrointestinal infections.

## Introduction

Protection of mucosal surfaces against colonization and possible entry and invasion by microbes is provided by a combination of constitutive, non-specific substances (mucus, lysozyme, and defensins), and by specific cellular and molecular immune mechanisms including secretory immunoglobulins (SIgs) (1, 2). *In vivo*, experimental and clinical resistance to infection can be correlated with specific SIgA antibodies (Abs) serving as an immunological barrier at mucosal surfaces (3, 4). They are produced by plasma cells in the basolateral part of the epithelium as dimeric molecules thanks to the presence of the J-chain. IgA are transported across the epithelial barrier to the lumen by a specific receptor that after cleavage remains linked to IgA and forms the secretory component (SC). Association of dimeric IgA with SC leads to SIgA generation. It is thought that agglutination, immobilization, and neutralization of pathogens at mucosal surfaces is facilitated by the multivalency of SIgA (5, 6) and the presence of the SC (7). IgM offers a higher multivalency due to its pentameric structure. Presence of the J-chain on IgM allows also for the binding of SC and the generation of SIgM that are released in the lumen similarly to SIgA. In patients with IgA-deficient individuals, SIgM seem to serve as a surrogate of SIgA (8) acting via a similar protective mechanism (9). However, its assessment *in vivo* is missing.

The lack of effective vaccines against several infectious agents, the emergence of new pathogenic strains, and problems associated with antibiotic resistance, has led to a renewed interest for passive immunization consisting of the exogenous delivery of Abs as prophylactic and therapeutic agents. In emergency situations that cannot wait for a vaccine to induce protective immunity, direct intervention with biologically active Ab molecules to the affected site represents the basis for topical immunotherapy (10). Such a strategy relying on intravenous injection of polyreactive IgG preparations (IVIg) has already proven of high value in the case of severe systemic infections (11). Because neutralization of microbes at mucosal surfaces is largely mediated by SIgs, application along the gastrointestinal tract of purified plasma-derived polyreactive IgA and IgM reconstituted in SIgs (12) (SCIgA/M) deserves appraisal as an anti-pathogen approach. Such preparations are derived from a pool of plasma from thousands of donors and therefore contain both natural polyreactive Abs known to interact with/control pathogenic micro-organisms (13–15) and a repertoire of polyspecificities to pathogens due to the natural history of infections that the donors have been exposed to (16).

We have previously established that secretory-like IgA and IgM (SCIgA/M) had the capacity to interact with the enteropathogen *Salmonella enterica* Typhimurium and promoted the formation of large aggregates of this particular bacterium (17). Upon oral administration of immune complexes, immune exclusion of the pathogen by SCIgA, and SCIgM Abs resulted in reduced local infection in the gut and diminished systemic dissemination (17). Fulfillment of these essential prerequisites indicates that the exogenously delivered Ab molecules exhibit the same functional features as locally secreted endogenous Abs in the harsh gut environment.

Using a stringent experimental mouse model of *S. enterica* Typhimurium (St) gut infection, we demonstrate that two prophylactic oral administrations of SCIgA/M given 8 and 24 h prior to infection with a lethal dose of St reduces tissue bacterial load and mortality rate more efficiently than polymeric IgA/M or IgG. In the more demanding therapeutic setting, one single dose of SCIgA/M proved efficacious when delivered orally 1 or 8 h post-infection. Intrinsic stability and proper mucosal tethering of SCIgA/M caused the difference in efficacy, and marks this particular molecular form as that with optimal functionality for oral immunotherapeutic intervention.

## Materials and Methods

### Preparation of Human Plasma-Derived Abs

Purified human plasma-derived IgA/M and SCIgA/M were prepared as published (17). Privigen (CSL Behring) was used as the source of purified human plasma-derived IgG. For visualization *in vivo*, Ab preparations were labeled with indocyanin 5 (Cy5) fluorescent dye (Innova Biosciences Ltd).

### Bacterial Strain and Culture Conditions

SL1344 [a streptomycin-resistant virulent strain of *S. enterica* serovar Typhimurium (18); abbreviated St all along the paper] was grown in Luria-Bertani broth containing 90 μg/ml streptomycin (Sigma-Aldrich) and used at mid-log growth phase. Bacterial density was determined on the basis that 1 OD_600nm_ corresponds to 9.5 × 10^8^ CFU/ml. The inoculation dose was verified by plating 10-fold serial dilutions on agar plates.

### Mice

Four week-old female BALB/c mice were obtained from Charles River Laboratories (L'Arbresle, France) and used at the age of 7–8 weeks. They were housed in the animal facility of the Lausanne University State Hospital under standard conditions. All experiments were approved by the State Veterinary Office, Lausanne, Switzerland (permit number VD2880) and performed in strict accordance to the guidelines of the animal experimentation law (SR 455.163) of the Swiss Federal Government.

### Ligated Intestinal Loops and Analysis of Tissue Sections

Ligated intestinal loops were prepared (19), and 100 μl of a solution containing 10 μg of Cy5-labeled SCIgA/M, IgA/M, or IgG were delivered into the lumen. Mice were sacrificed 6 h later (i.e., the maximal incubation time allowed by the State Veterinary Office), the intestinal segment was removed, fixed in 500 μl of PBS-4% paraformaldehyde (Fluka) for 2 h at 4°C, and further processed as described (20). Tissue sections were labeled with rabbit anti-mouse/human-MUC-2 IgG (1/50; Santa-Cruz Biotechnology), followed by AlexaFluor647-labeled goat anti-rabbit IgG (1/200; Life Technologies). Cell nuclei were stained with DAPI. Laser scanning confocal microscopy images were obtained using a Leica SP5 microscope in multi-track mode. Raw images were analyzed and processed with Imaris 8 software to generate 3D reconstitutions. One representative tissue section, out of at least 2 independent experiments (several sections per experiments), is displayed for each condition.

### Ab Degradation Assay

The *in vitro* degradation assay was performed as described (21). Detection of the reduced form of the heavy chain of each Ab mixture was performed by immunoblotting using goat anti-human alpha chain (1/1,000; Cappel), followed by HRP-labeled rabbit anti-goat Ab (1/4,000; Sigma-Aldrich), or biotinylated anti-human mu chain (1/1,000; KPL) or biotinylated anti-human gamma chain (1/1,000; Sigma-Aldrich) sera, followed by Extravidin-HRP (1/4,000; Sigma-Aldrich). The relative quantification of intact heavy chains following degradation as compared to the initial condition (0 h) has been performed with Fusion FX7 software (Vilber Lourmat). Displayed blots are examples of one particular experiment out of at least three that have been compiled for the quantification analysis shown in the **Table 2**.

For the *in vivo* degradation assay, 2 μg of unlabeled Abs in 20 μl of PBS were injected in a mouse ligated intestinal loop. After 6 h, mice were sacrificed, the loop's tissue and luminal content were collected and cut into small pieces. The samples were incubated in 50 mM HCl and Complete^TM^ protease inhibitor (Roche Applied Science) for 16 h at 4°C under gentle shaking, then centrifuged for 10 min at 2,350 × g. The clear supernatant was collected and stored at −20°C prior to use. To ensure equivalent samples loading, the concentration of Abs was determined through measurement by ELISA of stable anti-human kappa chain signal (21). SDS-PAGE and immunoblot analyses were performed as for the *in vitro* assay. The relative quantification of degraded heavy chains signals as compared to the total heavy chain signals has been performed with Fusion FX7 software (Vilber Lourmat). Displayed blots are examples of one particular experiment out of at least three that have been compiled for the quantification analysis.

### Oral Infection of Mice and Passive Administration of Ab

Mice were orally infected with 2 × 10^7^ (to assess bacterial entry in prophylactic and therapeutic settings), 2 × 10^6^ (to assess bacterial entry in therapeutic settings), and 2 × 10^6^ (to determine survival in prophylactic and therapeutic settings) St using a round tip stainless steel needle (22) (Table 1). In the prophylactic setting, 10 mg of plasma-derived polyreactive SCIgA/M, IgA/M, or IgG were orally administered 24 h and 8 h prior to bacteria infection. In the therapeutic setting, 10 mg of SCIgA/M were orally administered 1 h or 8 h post-infection. Bacterial loads in Peyer's patches, mesenteric lymph nodes, spleen, and liver were assessed 6 days post-infection by plating serial dilutions of tissue lysates (22). Weight of mice and disease score (23) based on fur ruffling, activity, posture, eye/nose discharge, and aspect of feces were recorded on a daily basis. A compilation of data from three (bacterial entry) or two (survival) independent experiments is displayed.

**Table 1:** Oral infection of mice and passive administration of Abs.

**PROPHYLACTIC SETTINGS**				
**Bacterial infection assay**				
Mock-treated	PBS	150 μl	2 × 10^7^ St	100 μl
Ab-treated	IgA/M/SCIgA/M/IgG	150 μl	2 × 10^7^ St	100 μl
**Mouse survival assay**				
Mock-treated	PBS	150 μl	2 × 10^6^ St	100 μl
Ab-treated	IgA/M/SCIgA/M/IgG	150 μl	2 × 10^6^ St	100 μl
**THERAPEUTIC SETTINGS**				
**Bacterial infection assay (treatment at + 1 h only)**				
Mock-treated	PBS	150 μl	2 × 10^7^ St	100 μl
Ab-treated	SCIgA/M	150 μl	2 × 10^7^ St	100 μl
**Bacterial infection assay (treatment at + 1 h or + 8 h)**				
Mock-treated	PBS	150 μl	2 × 10^6^ St	100 μl
Ab-treated	SCIgA/M	150 μl	2 × 10^6^ St	100 μl
**Mouse survival assay**				
Mock-treated	PBS	150 μl	2 × 10^6^ St	100 μl
Ab-treated	SCIgA/M	150 μl	2 × 10^6^ St	100 μl

### Histological Analysis of Intestinal Tissues

At sacrifice, 1-cm sections of jejunum were collected, fixed in paraformaldehyde, and cut into 5 parts before embedding in paraffin. Seven micrometer sections were then prepared and stained with hematoxylin and eosin to assess tissue integrity. At least 10 tissue sections per animal were observed with an Axio Imager Z1 microscope (Zeiss). One representative tissue section is displayed for each condition.

### Statistical Analysis

Statistical analysis was performed using Prism software (GraphPad Software, Inc., La Jolla, CA). Bars represent median of each experimental group. The unpaired, non-parametric Mann–Whitney test, or the Kruskal–Wallis test, corrected with Dunn's test for multiple comparisons, was applied to compare experimental groups with the “no Ab” condition, or SCIgA/M vs. IgA/M. For survival experiments, the Kaplan-Meier curves were calculated and a log-rank test was used to compare the survival curves. ns, non-significant; ^*^p < 0.05; ^**^p < 0.01; ^***^p < 0.001; ^****^p < 0.0001.

## Results

### Plasma-Derived Polyreactive (SC)Ig/AM Is Stable in the Intestinal Environment

**Figure 1:**
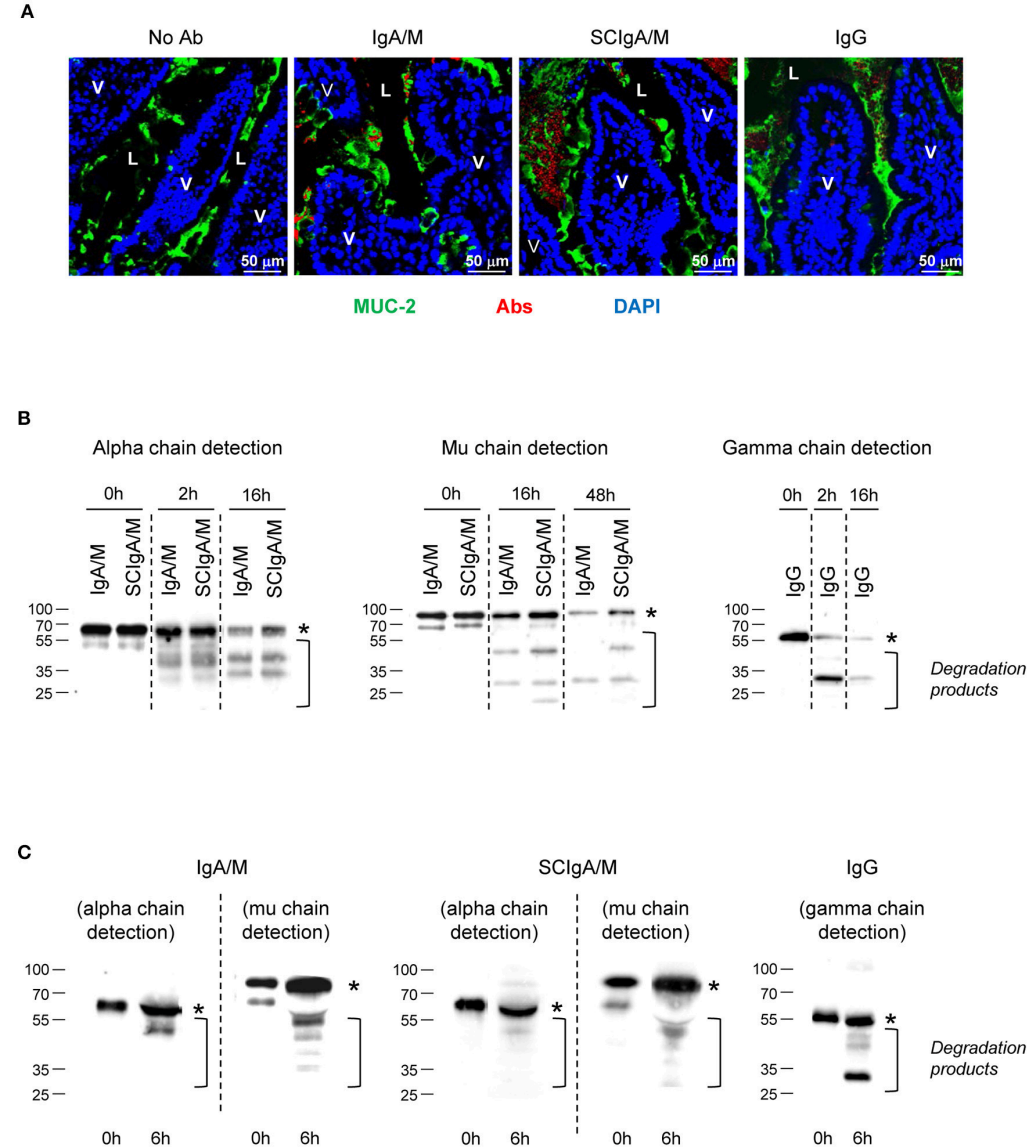
Location and stability of Ab preparations in the gastrointestinal tract. **(A)** Tissue sections prepared from mouse ligated intestinal loops 1.5 h post-administration with either Cy5-labeled IgA/M, SCIgA/M, or IgG appearing in red. Sections were further stained with anti-MUC-2 antiserum and DAPI to detect mucus and cell nuclei prior to analysis by laser scanning confocal microscopy. Examples of typical results of more than five samples are shown. L, Lumen. Magnification, x 40 for all panels. **(B)** *In vitro* digestion patterns observed at three time points of IgA/M, SCIgA/M, and IgG incubated with intestinal washes, as assayed by immunodetection of the respective heavy chain under reducing conditions. In **(B,C)**, the position of the intact alpha, mu, and gamma chains is indicated by an asterisk. **(C)** IgA/M, SCIgA/M, or IgG incubated for 6 h in a ligated intestinal loop were recovered as described in Materials and Methods, and analyzed as under **(B)**.

The prerequisite for efficient humoral protection in the gut is the ability of the Ab to interact with the mucus to limit its elimination, and to resist to degradation by local proteases. To assess bio-distribution, Cy5-labeled SCIgA/M, IgA/M, or IgG Ab preparations were injected into a ligated intestinal loop and incubated for 6 h prior to section preparation and staining for MUC-2, a constituent of the mouse intestinal mucus. Analysis by laser scanning confocal microscopy revealed numerous SCIgA/M-corresponding bright red fluorescent spots embedded within the MUC-2-positive areas overlying the epithelial cell layer (Figure 1A). Less numerous and much weaker signals were observed following administration of IgA/M or IgG in the ligated intestinal loop. This indicates a reduced capacity of these two Ab preparations to anchor in the mucus layer, consistent with the essential role of SC in tethering polymeric Igs within mucus (7), and with the higher susceptibility to protease-mediated degradation for non-secretory-like Igs in such an environment.

To address this possibility, the stability of the same set of Abs was examined by testing their sensitivity to mouse intestinal washes. After 2 h of incubation in intestinal washes, immunoblots performed in reducing conditions indicated the presence of intact alpha chain and some degradation products (Figure 1B and Table 2). After overnight incubation, more intact alpha chain signals were recovered for SCIgA/M preparations as compared to IgA/M, consistent with the protective role ensured by SC (21). The lesser sensitivity of mu chain to proteases in intestinal washes (24) imposed to prolong digestion for up to 48 h; the presence of SC in SCIgA/M reduced the conversion of the mu chain into degraded species (Figure 1B and Table 2). In contrast, when IgG was analyzed, the gamma chain was already largely degraded after as few as 2 h, and almost no signal could be detected after 16 h. Experiments performed *in vivo* in ligated intestinal loops showed that the degradation of alpha and mu chains from SCIgA/M and IgA/M was very partial, with slightly less degradation for SCIgA/M. The proportions of degraded materials were 43.9 ± 12.3% of alpha chain and 25.4 ± 16.9% for mu chain (SCIgA/M) as compared to 66.4 ± 20.0% for alpha chain and 53.6 ± 6.3% for mu chain (IgA/M). Analysis of IgG resulted in the appearance of a signal well below the expected molecular weight of native gamma chain (Figure 1C), representing 57.5 ± 11.7% of gamma chain. Due to the digestion time limited to 6 h for *in vivo* experiments (see Materials and Methods), the presence of SC in SCIgA/M led to a limited advantage in stability over IgA/M.

**Table 2:** Quantification of intact heavy chains in the *in vitro* degradation assay.

	**Alpha chain**		**Mu chain**		**Gamma chain**	
	**2 h**	**16 h**	**16 h**	**48 h**	**2 h**	**16 h**
IgA/M	47.2 ± 9.2%	23.8 ± 11.9%	38.6 ± 18.1%	27.3 ± 11.1%	n.d.	n.d.
SCIgA/M	64.3 ± 24.8%	26.8 ± 9.8%	47.9 ± 10.7%	38.0 ± 10.5%	n.d.	n.d.
IgG	n.d.	n.d.	n.d.	n.d.	35.4 ± 0.5%	11.4 ± 1.5%

*n.d., not done*.

Together, these data demonstrate that after administration in the intestinal environment, the SCIgA/M preparation, as compared to IgA/M and IgG, combines the most optimal anchoring and stability properties, two parameters that have been associated with protective efficacy in the gut.

### Protection Against *Salmonella* Infection Is Best Mediated by Prophylactic Oral Administration of SCIgA/M

**Figure 2:**
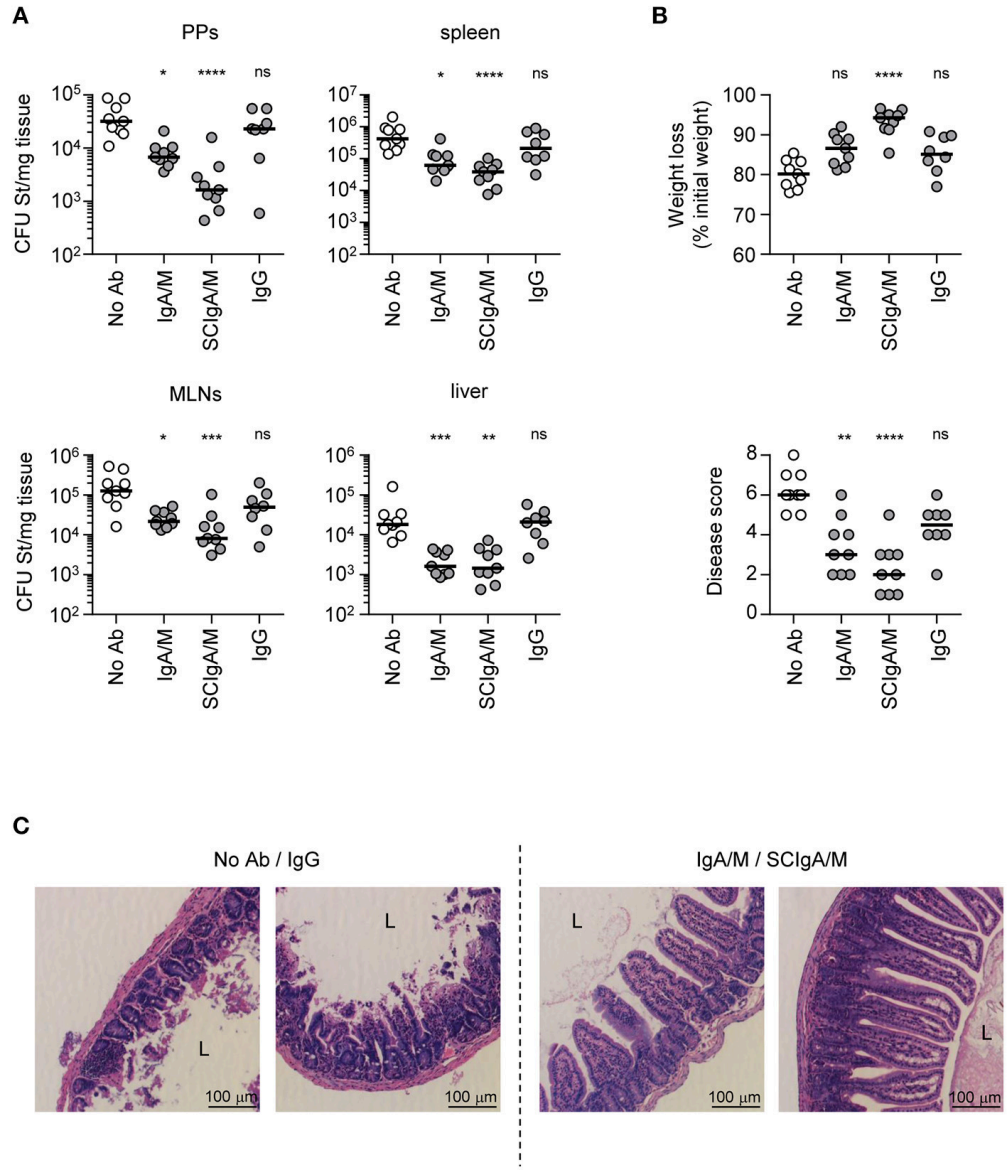
Prophylactic administration of SCIgA/M preparation limits St entry and reduces symptoms. **(A)** Bacterial counts measured in Peyer's patches (PPs), mesenteric lymph nodes (MLNs), spleen, and liver 6 days post-infection by oral administration of 2 × 10^7^ St into mice prophylactically treated with Ab preparations. **(B)** Weight loss and disease score (see section Materials and Methods) determined at day 6 post-infection as a function of the Ab preparation administered prophylactically. Each panel compiles the results of three independent experiments, conducted with 3 mice per group. **(C)** Histologic examination (hematoxylin and eosin staining) of intestinal tissue sections obtained from infected mice 6 days post-administration with a single dose of either 2 × 10^7^ St alone, or in the presence of prophylactically applied Ab preparation. Typical patterns for “No Ab”/IgG (left) and IgA/M/SCIgA/M (right) representative of more than 10 sections. L, Lumen; V, Villus. Magnification, x 20 for all images. ns, non-significant; *p < 0.05; **p < 0.01; ***p < 0.001; ****p < 0.0001.

To test the biological activity of SCIgA/M, as well as the importance of SC in the preparation, prophylactic oral administration of 10 mg of SCIgA/M, IgA/M and IgG (as control) was performed at 24 h and 8 h before intragastric inoculation with 2 × 10^7^ CFUs of St. Mice were kept for 6 days post-infection, and the protective efficacy of the Abs was assessed at this time by measuring the local intestinal and distant systemic bacterial load. Prophylactic passive immunization with SCIgA/M turned out to be the most potent at reducing the bacterial load in Peyer's patches, mesenteric lymph nodes, the spleen, and the liver of infected mice (Figure 2A). Physiological symptoms of infection including weight loss and disease score were best reduced upon SCIgA/M administration (Figure 2B). IgA/M showed some efficacy, as reflected by a significantly weaker disease score in comparison with untreated-infected mice. A direct comparison between SCIgA/M and IgA/M demonstrated a clear trend toward a better protective effect of the former (*p* = 0.0040 for Peyer's patches, *p* = 0.0625 for mesenteric lymph nodes, *p* = 0.0625 for the spleen, *p* = 0.8633 for the liver, *p* = 0.0019 for weight loss and *p* = 0.1110 for disease scores). For all parameters assessed, prophylactic treatment with IgG yielded no sign of improvement of the animal's health status at day 6 post-infection (Figure 2B).

St infection and its associated inflammatory response are known to damage the intestinal epithelium, leading to additional alteration of the gut function. Consistent with its capacity to limit infection, prophylactic administration of SCIgA/M, and to a lesser extent IgA/M, contributed to largely maintain the architecture of the intestinal tissue 6 days post-infection. As depicted in Figure 2C, histological analysis of intestinal tissue showed preserved integrity of the epithelium, with only limited shortening of the villi. In comparison, untreated-infected or IgG treated-infected mice exhibited important tissue damages as reflected by the massive destruction of the intestinal villus structure (Figure 2C).

### SCIgA/M Delays Disease Progression and Improves Survival of Infected Mice

**Figure 3:**
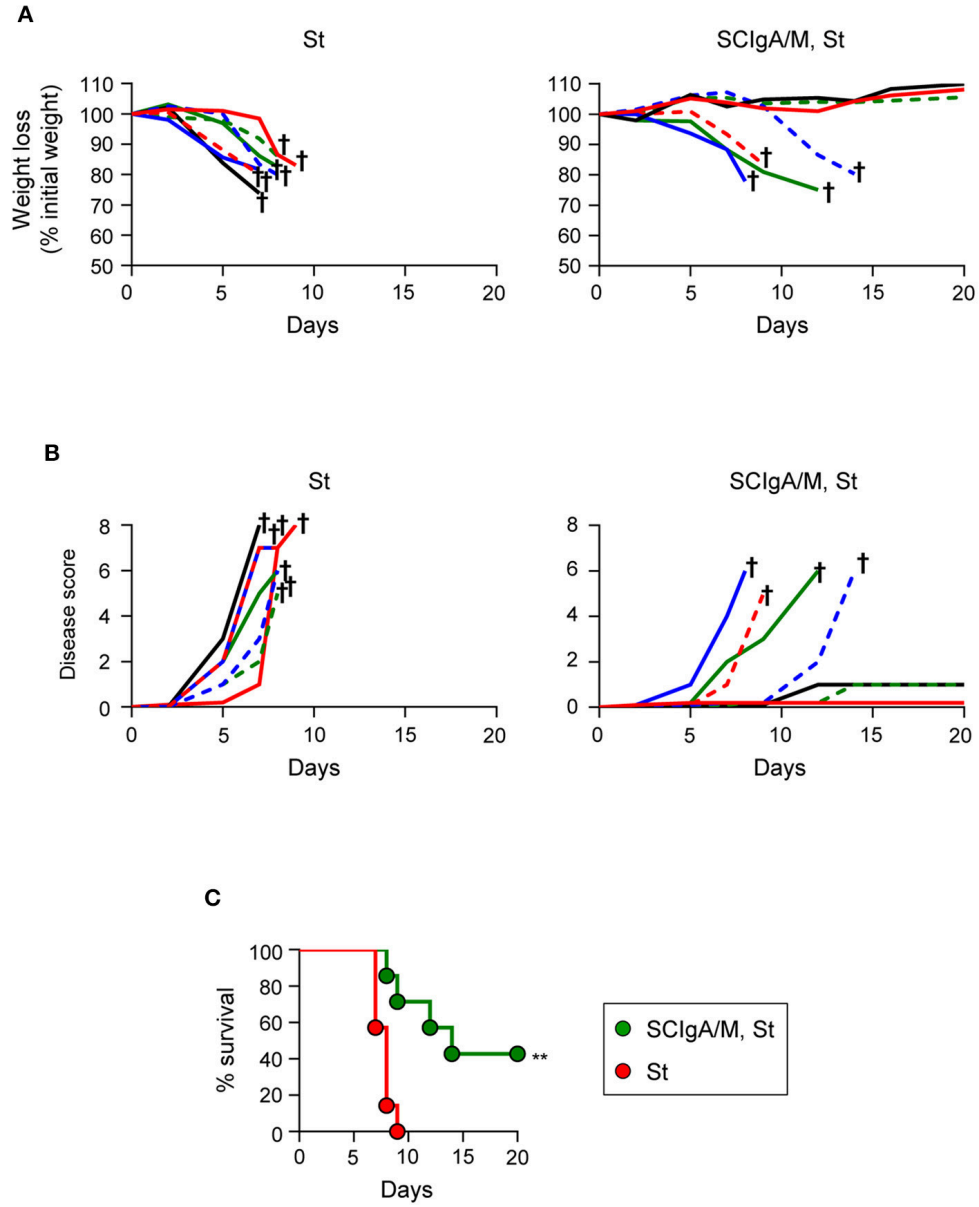
Prophylactic administration of SCIgA/M preparation promotes mouse survival. Time-dependent follow-up of mice infected with 2 × 10^6^ St post-prophylactic treatment (24 and 8 h) with oral delivery of the SCIgA/M preparation. Each depicted curve represents one individual mouse analyzed for weight loss **(A)** and disease scores **(B)**. Sacrifice (depicted by a †) of mice having lost ≥20% of their initial weight was done to comply with the Veterinary Office's permit to conduct animal experiments. Each panel compiles the results of 2 independent experiments. **(C)** Survival curves resulting from the compilation of all mice in either the St alone group (red circles) or the prophylactically treated (SCIgA/M, St) group (green circles). **p < 0.01.

The correlation between the residual bacterial load and the possible effect on the survival is important to validate protection. To address this issue, the experimental focus was put on animals having received prophylactically the best protective candidate Ab, i.e., SCIgA/M at a dose of 10 mg given orally 24 h and 8 h prior to infection with 2 × 10^6^ St. In contrast to control animals rapidly losing weight and reaching a high disease score (Figures 3A,B), 5 out of 7 treated-infected mice showed a delay in symptom progression. When looking at the survival rate, all untreated mice died within 9 days post-infection (Figure 3C), while 3 out of 7 survived after having received SCIgA/M. Overall, prophylactic passive administration of the SCIgA/M preparation has the ability to protect mice from subsequent oral infection by lethal doses of St via multiple modes of action including reduction of the bacterial load in tissues, maintenance of the epithelium architecture, and delay of disease progression or complete healing.

### Therapeutic Oral Administration of SCIgA/M Protects Against *Salmonella* Infection

**Figure 4:**
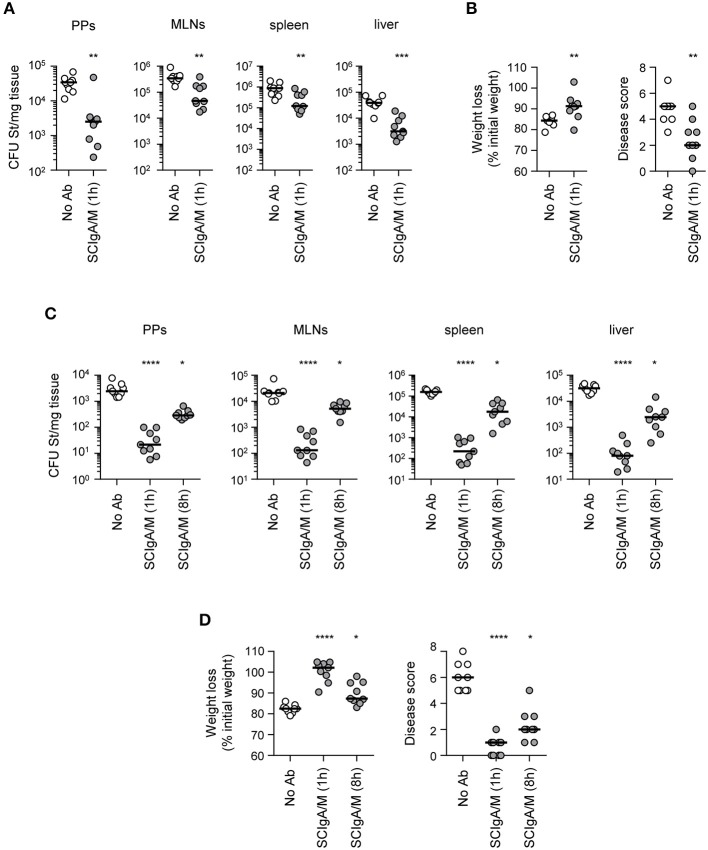
Therapeutic administration of SCIgA/M limits St entry and reduces symptoms **(A)** Bacterial counts measured 6 days post-infection with 2 × 10^7^ St in Peyer's patches (PPs), mesenteric lymph nodes (MLNs), spleen, and liver from mice orally administered SCIgA/M 1 h after oral infection. **(B)** Weight loss and disease scores (see Materials and Methods) determined at day 6 post-infection of mice treated therapeutically with SCIgA/M as in **(A)**. **(C,D)** Same experiments as in **(A,B)** with infection performed with 2 × 10^6^ St, followed by subsequent therapeutic oral administration of SCIgA/M 1 or 8 h after oral infection. Each panel compiles the results of 3 independent experiments, conducted with 3 mice per group. ns, non-significant; *p < 0.05; **p < 0.01; ***p < 0.001; ****p < 0.0001.

**Figure 5:**
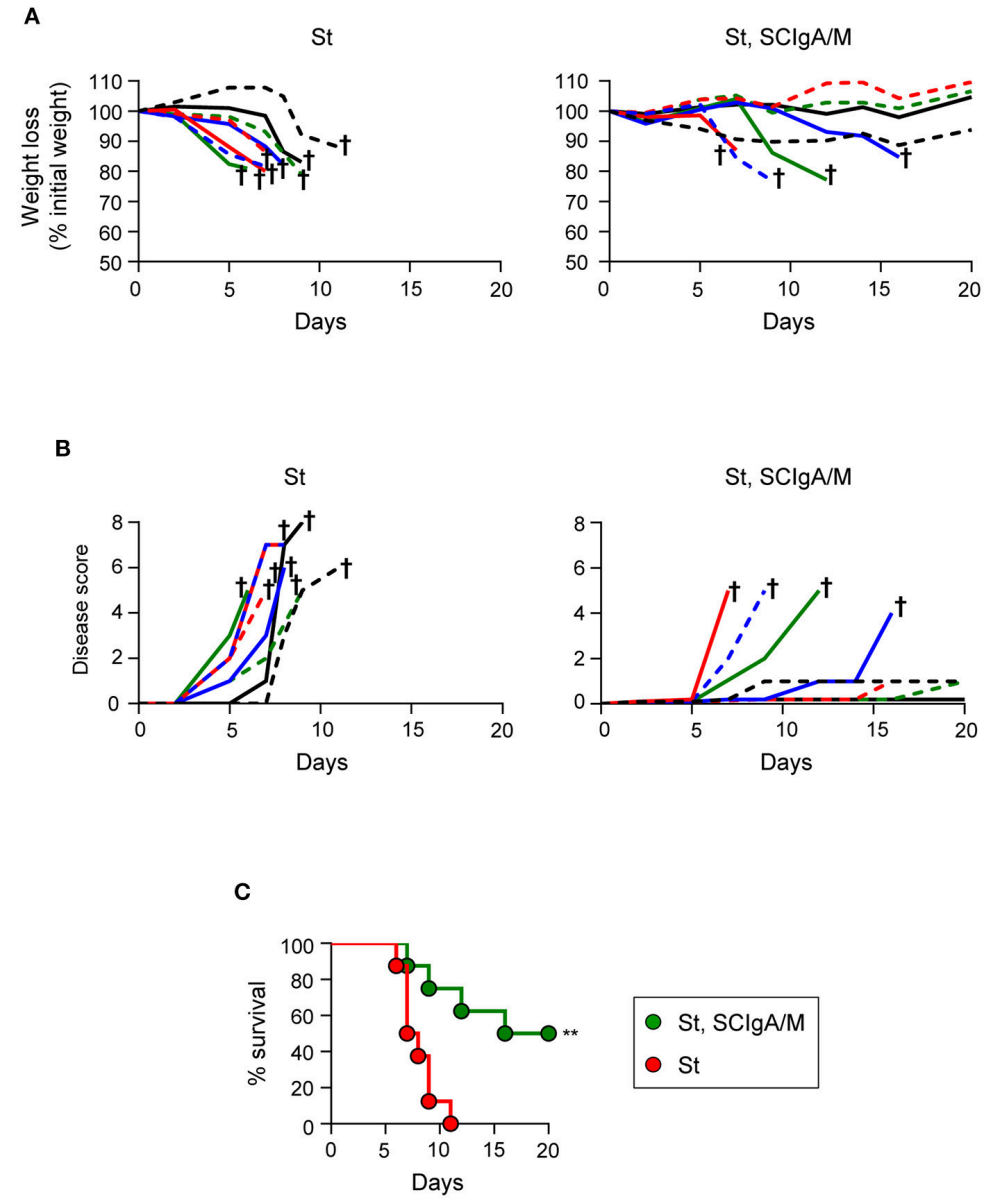
Therapeutic administration of SCIgA/M preparation promotes mouse survival Time-dependent follow-up of mice infected with 2 × 10^6^ St prior to therapeutic treatment carried out 8 h post-infection with a single dose of the SCIgA/M preparation. Each depicted curve represents one individual mouse analyzed for weight loss **(A)** and disease scores **(B)**. Sacrifice (depicted by a †) of mice having lost ≥20% of their initial weight was done to comply with the Veterinary Office's permit to conduct animal experiments. Each panel compiles the results of 2 independent experiments. **(C)** Survival curves resulting from the compilation of all mice in either the St alone group (red circles) or the therapeutically treated (St, SCIgA/M) group (green circles). **p < 0.01.

The promising results obtained in the prophylactic setting prompted us to assess the therapeutic protective efficacy of SCIgA/M. When 2 × 10^7^ St were used to orally infect mice, administration of 10 mg SCIgA/M 1 h later was able to significantly reduce the bacterial load in mice recovered at day 6 post-infection in both intestinal (Peyer's patches and mesenteric lymph nodes) and systemic (spleen and liver) tissues (Figure 4A). Weight loss and disease score of treated mice were less pronounced as compared to untreated animals (Figure 4B). However, oral treatment performed 8 h post-infection did not result in reduction of the infection (data not shown), most likely due to the amount of bacteria that had rapidly moved across the epithelium prior to Ab treatment. We thus sought to assess the protective efficacy of therapeutically applied SCIgA/M with 10-fold less St, a dose that remains lethal between days 9 and 11 (see Figures 3C, 5A). Initial oral infection with 2 × 10^6^ St followed by oral delivery of 10 mg SCIgA/M 1 h later largely reduced the number of St 6 days post-infection in either local or systemic compartments (Figure 4C). Under these experimental conditions, delaying the time of SCIgA/M administration to 8 h post-infection kept diminishing the bacterial load significantly (Figure 4C). This translated into improved status of the treated mice as compared to control animals, resulting in less weight loss and a lower disease score (Figure 4D).

The protective efficacy of a single dose of SCIgA/M assessed at day 6 post-infection led us to hypothesize that therapeutic application of these polyreactive Abs may have a positive effect on the survival of mice post-infection. We therefore orally infected mice with 2 × 10^6^ St, and applied 10 mg of SCIgA/M 8 h later; the weight, the disease score and the survival of animals were subsequently recorded for 20 days. In the untreated control group, mice rapidly lost weight (Figure 5A) and demonstrated signs of disease (Figure 5B), leading to the death of all mice at day 11 post-infection (Figure 5C). In contrast, 4 out of 8 animals therapeutically treated with one oral administration of 10 mg SCIgAM remained healthy for up to 20 days after infection. Protected mice did not lose weight nor exhibit signs of disease more pronounced than fur ruffling (Figure 5C). The other half died from the infection, but displayed a delay in both weight loss and the disease score's evolution, as compared to control mice, hence leading to prolonged survival.

Altogether, our data demonstrate that a single oral therapeutic administration of polyreactive SCIgA/M at a time when St infection is already ongoing entails the reduction of the intestinal and systemic bacterial load and of the symptoms associated with infection. Such a reduced invasiveness results in too little colonization to cause fatal infection in 50% of infected mice, comparing with 100% mortality in the absence of therapeutic SCIgA/M.

## Discussion

For thousands of generations, mammals have exploited the benefits of transferring colostrum- and breast milk-derived Abs to newborns to ensure protection early in life via the large repertoire associated with the mother's previous exposure to environmental pathogens (25–28). This evolution-related way of providing protective Abs finds its current application in systemic passive immunotherapy with polyclonal blood-based, or monoclonal Abs, mostly IgGs (29–31). While efficacy has been demonstrated in several human clinical trials at the systemic level (32–36), examples of successful protection of mucosal surfaces by local application of purified IgA (37, 38) or SIgA (39, 40) are not plethora in the literature. Besides, such studies were performed with monoclonal Abs only, while examples with SIgM are missing. The rationale of our strategy, which relies on the use of plasma-derived polyreactive SCIgA and SCIgM delivered by the oral route, is based on the close clonal relationship between mucosal and systemic plasma cells (41). In addition, circulating IgA^+^ plasma cells originate from mucosal immune inductive sites and disseminate systemically (42), resulting in the production of polymeric IgA in the blood. In support of this, we have observed that such antibody molecules recovered from plasma samples bind to SC (12). This ensures efficient recognition and control of enteropathogens both at their site of entry and following systemic dissemination. These two important features expected for mucosal passive immunization are observed following prophylactic or therapeutic intervention with SCIgA/M.

Exogenous mucosal delivery along the gastrointestinal tract implies that the optimal molecular form of the Ab is administered, i.e., SIgs. In support of their use for local passive immunization, natural SIgA molecules display high *in vitro* stability upon exposure to intestinal washes rich in proteases (21). Consistent with this, the current study shows that the lack of SC reduces the stability of either plasma-derived IgA or IgM *in vivo* and precludes proper anchoring within mucus. Such biochemical features are crucial to ensure optimal functionality of the SCIgA/M preparation toward enteropathogens, as this is demonstrated inhere in prophylactic and therapeutic tratments against *Salmonella*. In this context, control of the infection is known to occur via immune exclusion which is based on agglutination, enchained growth or both depending on the local concentration of bacteria in the infected intestine (17, 43). Interestingly, even though endogenous vaccine-induced high-avidity IgA Abs seem highly potent at controlling *Salmonella* infection (43), exogenous delivery of polyreactive SIgs displays a promising level of protection. When assessed individually, we found that purified SCIgA or SCIgM significantly decreased the weight loss and disease score in infected animals, with SCIgM showing the best performance (GB, unpublished data). This suggests that polyreactive SIgM contributes to mucosal protection, and provides a molecular explanation as to why the Ab can compensate for SIgA in IgA-deficient individuals (44).

In contrast to maternal IgG possibly protected in the colostrum/milk environment, passive delivery along the oral route of this isotype in a purified form did not show any positive impact on bacterial entry and dissemination. Based on our results, the absence of protection after oral passive administration of IgG can be attributed to the inadequate immunoglobulin isotype and/or the instability of the Ab in the gut, further arguing in favor of the use of polymeric and optimally secretory Igs. A logical development of the study that already shows efficacy after a single-dose in the therapeutic setting will involve multiple experimental refinements requiring that different doses of both the Ab and *Salmonella* be tested with various kinetics of administration; we find it fair to say that such a demanding development is beyond the scope of this proof-of-concept study.

Due to their intrinsic polyreactivity toward a broad range of antigens, human plasma-derived polymeric Igs are somehow “ready-to-be-used,” in contrast to humanized or chimeric specific monoclonal Abs, which may need to be combined to cover a sufficient diversity of recognition patterns. Another drawback, selection pressure on a particular bacterial antigen by repeated administration of a monoclonal Ab may favor mutation on one hand, and give growth advantage to other co-infecting microbes on another hand. While the technology to ensure production of monoclonal IgG of defined specificity is well-established, generation of polymeric IgA/IgM-secreting clones is not an obvious task and may turn out to be pricy. In contrast, the identification of modern biotechnology-based processes allows the recovery of hundreds of gram amounts of biologically active SIg molecules. For all these reasons, plasma-derived IgA/M molecules thus appear a relevant alternative for future human applications. Further, features including natural pattern of glycosylation will not induce possible inhibitory human anti-mouse Ab responses, in particular along the gastrointestinal tract known to be a tolerance-prone environment. The absence of overreaction toward non-self proteins such as biotechnology-derived pharmaceuticals will thus not represent a problem with orally administered SCIgA/M. Moreover, the lack of activation of the complement cascade by SIgA (45, 46), restriction of both SIgA and SIgM in the intestinal lumen, and local complement regulatory proteins (47) should ensure absent or strongly reduced inflammation in the gut. In addition, naturally existing plasma-derived polyreactive Abs have a very low risk of technological failure during the development process, and problems associated with species matching for human application can be excluded. Another obvious advantage lies in the intrinsic capability of mucosally applied Ab preparations to neutralize the pathogen at a very early stage of infection, or even prior to the initiation of the infection. Data presented in this study bring valuable elements as to the Ab isotypes, which need to be delivered to remain active when facing a massive infection along the gastrointestinal tract. Finally, although addition or recombinant SC necessary for optimal function will have to be considered, its production via CHO cells does not represent a bottleneck anymore.

The natural protection offered by passive immunization has already provided the physicians with the opportunity to envisage treatments against mucosal infectious agents with Ab-enriched milk, serum, or egg yolk preparations (48). The emergence of bacterial strains resistant to multiple antibiotics and the major progresses made in Ab engineering give strong arguments to thrive implementation of passive immunization. The present study supports the notion that plasma-derived polyreactive SCIgA and SCIgM may contribute to fulfill this health need.

## Author Contributions

BC, CV, AS, and GB designed the experiments. JM and GB performed research and analyzed data. ML provided reagents. BC, CV, and GB wrote the manuscript. All authors have read, critically revised, and approved the final manuscript.

### Conflict of Interest Statement

ML, CV, and AS are employees of CSL Behring AG. The remaining authors declare that the research was conducted in the absence of any commercial or financial relationships that could be construed as a potential conflict of interest.

## Abbreviations

IgA/M: plasma-derived polyreactive IgA and IgM
SIgs: secretory immunoglobulins
SC: secretory component
SCIgA/M: secretory-like IgA/M
St: *Salmonella enterica* Typhimurium strain SL1344.
